# Investigating the input power quality of multi-pulse AC-DC power converter fed induction motor drives

**DOI:** 10.1016/j.heliyon.2022.e11733

**Published:** 2022-11-29

**Authors:** Nazmul Islam Nahin, Mahdee Nafis, Shuvra Prokash Biswas, Md. Kamal Hosain, Pranta Das, Safa Haq

**Affiliations:** Electronics & Telecommunication Engineering, Rajshahi University of Engineering & Technology, Rajshahi, Bangladesh

**Keywords:** Multi-pulse, AC-DC power converter, Induction motor drive, Supply current, Total harmonic distortion

## Abstract

Optimization of supply current total harmonic distortion (THD) of multi-pulse AC-DC power converter fed induction motor drive (IMD) is always a challenging issue. Higher amount of supply current THD degrades the input power quality of IMD. The supply current THD should be controlled in such a way so that it adheres the power quality standard of IEEE-519. With the increase of the pulse number of multi-pulse AC-DC converter, supply current THD increases. In this work, an investigation has been carried out on 6-pulse, 12-pulse, 18-pulse, and 24-pulse AC-DC power converters based IMD. A thorough analysis of input current profile, THD, dynamic responses including stator current, speed, torque profile of the induction motor are highlighted in this work with the various load perturbation conditions. This work will provide a message to the industrial community about the proper selection of AC-DC power converter for IMD application considering power quality and circuit configuration issues. All the investigating works are conducted in MATLAB/Simulink platform.

## 1 Introduction

In the recent years, AC-DC converters are extensively used in numerous applications such as variable frequency induction motor drives (VFIMD), high voltage DC transmission (HVDC), renewable energy conversion, control systems, aerospace, and in daily electrical appliances. They usually require DC power for operation, though some applications including AC drives require DC power at transitional phases. These AC-DC converters are usually known as the rectifier which are fed by three phase AC supply having 33 kV or 11 kV followed by a step down transformer for medium power applications [1]. However, these AC-DC converters suffer from poor input power quality, increased total harmonic distortion (THD), low input power factor, and increased DC voltage ripple. To remove these limitations, various methods have been devised in [2], [3], [4], [5]. Generally, filters are used to remove harmonics which can be categorized into active or passive. These filters are not economical, increase losses and circuit complexity. As a result, for improving the input power quality and reducing harmonics, multi pulse rectifiers (MPR) have obtained popularity due to their remarkable efficiency, high robustness and simple configuration. Moreover, multi-pulse rectifiers do not require any LC filters which remove the possibility of LC resonances and the common mode voltage produced by rectifiers are eliminated by the employment of phase shifting transformer. MPR utilizes the phase shifted transformer to lessen the harmonics at the input side and allows multiple rectifiers to operate in series or separately. In general, the pulse number determines the limiting capability of harmonics of MPR [6].

MPCs are designed for AC-DC conversion at a mature level with low THD at the ac main and reduced rippled dc output with unidirectional and bidirectional power flow for feeding loads varying from kilowatts to megawatts. Multi-pulse rectifiers consist of a set of six pulse rectifiers along with a phase-shifting transformer. MPRs are categorized according to the number of pulses and its output rectifier connection which can be either series, separated or parallel [7]. Apart from that, they can also be classified based on connections such as extended delta and double star [8], [9], zigzag [10], [11], [12], reduced rating autotransformers and T-connections [13], [14], [15]. Phase shifting transformer is utilized to design the MPRs which includes splitting the supply into a number of outputs, each of which is phase-shifted to the others at a suitable angle to reduce harmonics. Phase shifting transformers are employed to mitigate the current harmonics and withstand the overheating occurs due to harmonic injection [16]. Detailed winding turns design and analysis of phase shifted multi-pulse rectifier is discussed in [17], [18], [19]. Different configurations of MPRs are now studied to meet the criteria for proper applications by maintaining the input power quality.

The most common type of MPR is 6-pulse rectifier which is used in low power applications due to its simplicity and less circuit complexity. However, at full load condition, it's input power quality degrades massively and total harmonic distortion can be surpassed up to 100% with no harmonic filter. On the other hand, the 12-pulse rectifier is the most common among MPRs. Two 6-pulse diode bridge rectifiers combined with a multi-phase transformer construct a 12-pulse rectifier. Due to series connection of two 6-pulse rectifiers, the DC current ripple is quite low. The 12-pulse rectifier can impressively remove (6n ± 1) harmonics in the input line current but harmonics (12n ± 1) (where n is an integer) remain in the system. The 11^th^ and 13^th^ are the dominant harmonics [20]. Due to their low cost and reduced size, they are extensively used in numerous fields such as variable frequency drive (VFD), DC drives, electroplating and electric aircraft [21], [22]. Low input current THD and reduced dc voltage ripple are the feature of 18-pulse converter which makes it distinctive from the other converters. 18-pulse converter is constructed by connecting three 6-pulse rectifiers in series. One of the advantages of this converter is, it bypasses the input voltage harmonics and interphase components. It can effectively reduce dominant harmonics except for the 17^th^ and 19^th^ harmonics [23]. The 24 pulse converters are utilized in high power situations where the usage of numerous devices is allowed. It eliminates the majority of the dominant harmonics and provides input-side ac current that is almost sinusoidal and ripple-free dc output voltage [24]. As the number of pulse increases for the multi-pulse rectifier, the performance of the system also increases so as the system complexity.

Although the increasing of pulse number reduces harmonics at a certain limit, different strategies have also been established to reduce THD. Researchers have conducted various studies and devised numerous strategies so that the amount of THD in the input AC line current of AC-DC power converter can be reduced to a suitable range. Pulse width modulation (PWM) is one of the taken strategies to reduce THD. The PWM method uses constant amplitude pulses with variable duty cycles for each period. To adjust inverter output voltage and reduce harmonic content, the width of these pulses is controlled [25]. The PWM technique used in voltage source inverter (VSI) has a great impact on the performance parameters of IMD such as output voltage THD of an inverter, stator current THD, and motor torque ripple. These are the main performance parameters of IMD which greatly depend upon inverter switching technique [26]. The most notable PWM are sinusoidal PWM (SPWM) and conventional space vector PWM (CSVPWM). When compared to SPWM, CSVPWM and third harmonic injection PWM (THIPWM) result in higher line side voltage for the same DC bus voltage. Furthermore, for a given line voltage, CSVPWM and THIPWM produce less THD in motor currents and inverter output voltage than SPWM [27], [28], [29], [30]. Similarly, some advanced PWM strategies are also gained popularity due to their capability of reducing THD and losses such as third harmonic sixty-degree pulse width modulation (THSDPWM), sixty-degree PWM (SDPWM) and third harmonic trapezoidal PWM (THTRPWM) which are discussed in [31], [32], [33]. In addition, the hybrid PWM strategies, which are produced by merging CSVPWM and advanced bus clamping PWM (ABCPWM), have received attention due to their superiority in terms of increased efficiency, reduced switching losses, and minimized torque ripple over existing strategies [34]. Several torque ripple reduction approaches for AC-DC converter fed IMD have been discussed in [35], [36]. For low speed operation, torque ripple reduction of three level inverter fed IMD is found in [37]. PWM techniques have also gained popularity for reducing torque ripple. A new PWM method along with a novel rotor speed independent transformation can be found in [38]. Harmonics are mainly generated due to the constant on and off switching and connection of non-linear loads in the power system. In [39] improvement of THD performance on the grid side using a robust controller is studied. A comparative study control method for wind based energy system at grid side for reducing THD is given in [40]. However, the THD reduction at the inverter side focuses on the new controlling methods and different topologies. A brief review on multilevel inverter topologies is discussed here [41]. An efficient method of reducing of THD in cascaded H-bridge MLI can be presented in [42]. In [43], solar fed asymmetric MLI with reduced is presented. A cascaded MLI in a standalone PV system was suggested in [44], to reduce THD and produce high-quality power.

The main contributions of the paper are summarized as follows:

• Topologies such as 6-pulse, 12-pulse, 18-pulse, and 24-pulse system.
• To observe the line current and its harmonics profile for various configurations of the IMD.
• To observe the impact of different modulation techniques on the power quality parameters of IMD in terms of THD of supply voltage/current, distortion factor, power factor and dc-link voltage.
• To observe the dynamic response of IMD for 6-pulse, 12-pulse, 18-pulse, and 24-pulse converter systems in terms of stator current, speed and torque.
• To provide a clear comparative analysis among 6-pulse, 12-pulse, 18-pulse, and 24-pulse converter based IMD in terms of various power quality parameters and modulation techniques so that one can choose proper IMD configuration for specific application based on the study.

The performance of the IMD by utilizing 6-pulse, 12-pulse, 18-pulse, and 24-pulse converter systems has been investigated in detail in the study so that the industrial community can receive a clear message in the case of selecting proper configuration of IMD for specific industrial application.

## 2 Structure of induction motor drives

The multi-pulse rectifiers classified based on pulse number are discussed in this section. The topologies are elaborated in such a way that one can choose the right configuration for a specific application.

### 2.1 DC-link voltage estimation

In this work, a 2-level VSI is used to feed power to an induction motor. The voltage of the DC-link capacitor ($V_{dc}$) is found in [25]. The rectified voltage across the DC-link capacitor is given in (1),

(1) $$V_{dc}=\frac{V_{L-L}}{0.612a(L-1)}=\frac{420}{0.612\times 1\times 1}=686.27$$

where, $V_{L-L}$, *a* and *L* are the line-to-line voltage, modulation index, and inverter level respectively.

### 2.2 Transformer configuration

The elimination of lower order harmonics in multi-pulse rectifiers is achieved by proper arrangement of transformer winding. The harmonics generated in a system by multi-pulse rectifier can be found in (2),

(2) H=np±1

where, *H* is the harmonics generated by a rectifier, n=1,2,3…, and *p* is the pulse number. To eliminate these harmonics, the generalized phase-shifting angle is given in (3),

(3) $$\text{Phase shifting angle},\delta =\frac{360{}^{\circ}}{\text{No. of pulses}}$$

For 12-pulse rectifier, δ=360°/12=30°. Using Y-Y and Y-Δ configuration of transformer, this phase shift is easily obtained. For 18-pulse rectifier, δ=360°/18=20°. A phase-shifting transformer with a 20° phase difference between any two adjacent secondary windings can be used to achieve this. For the top, middle, and bottom secondary windings, the values of angle are 20°, 0°, and −20° respectively. For 24-pulse rectifier, δ=360°/24=15°. Zigzag transformer is used for providing 15° phase shifting angle as Y-Δ configuration is not suitable for it. −15°, 0°, +15° and 30° are the corresponding phase shifted angles for the four secondary transformers winding from each to each.

### 2.3 Description of the structure

**Figure 1:**
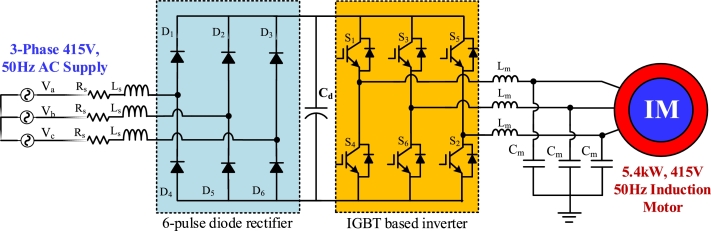
6-pulse AC-DC converter fed IMD.

**Figure 2:**
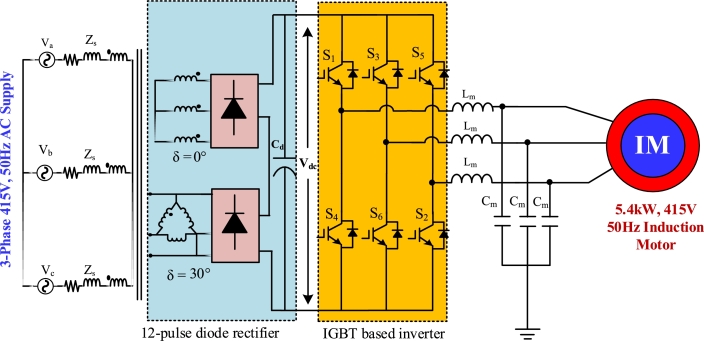
12-pulse AC-DC converter fed IMD.

**Figure 3:**
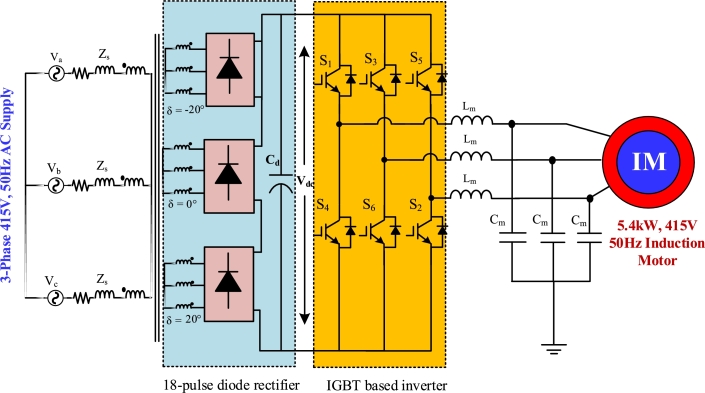
18-pulse AC-DC converter fed IMD.

**Figure 4:**
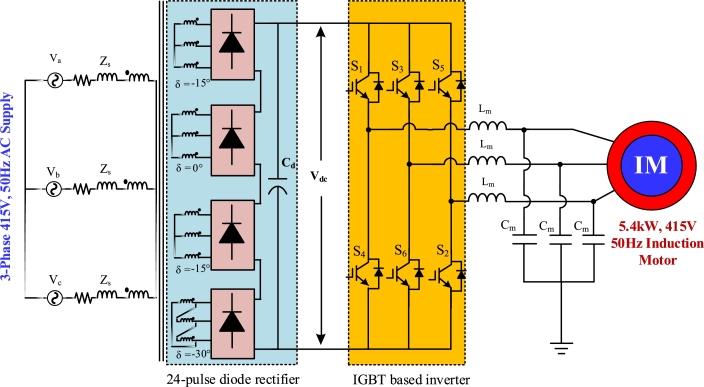
24-pulse AC-DC converter fed IMD.

Figure 1, Figure 2, Figure 3, Figure 4 depict the structures of multi-pulse AC-DC converter fed IMD. 6-pulse converter based IMD is shown in Fig. 1, where a traditional diode bridge rectifier is taking power from the 415 V, 50 Hz grid. The rectified DC voltage is filtered out through filter capacitor, Cd. Fig. 2 shows the 12-pulse system for feeding 5 kW induction motor. Two phase shifting transformers are utilized to feed power to the individual diode bridge units. The Y-Y transformer is shifted by 0°, whereas the Y-Δ transformer is shifted by 30° for 12-pulse. The filtered rectified voltage is used as the DC-link of the inverter. Similarly, in Fig. 3, the 18-pulse converter feeding power to IMD is shown where three phase shifted transformers are used to generate rectified voltage. The secondary winding of these three transformers is phase shifted by 20°, 0°, and −20°, respectively. In addition, four phase shifted transformers are used to construct 24-pulse converter which is depicted in Fig. 4. These four transformers windings are separated from each other by an angle of −15°, 0°, +15°, and 30°, correspondingly.

## 3 Various PWM techniques

SPWM, THPWM, THSDPWM, and CSVPWM are the most often used modulation techniques [26].

**Figure 5:**
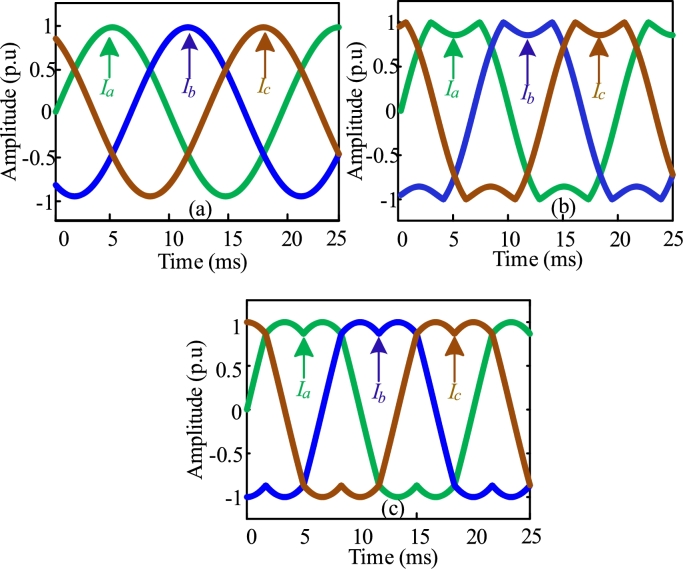
Modulating signals of: (a) SPWM, (b) THSDPWM, and (c) CSVPWM.

The modulating signals of SPWM, THSDPWM, and CSVPWM are shown in Fig. 5(a), 5(b), and 5(c), respectively. In PWM, a triangular carrier with a specific frequency is compared with the modulating signals and corresponding gate pulses are produced for bipolar insulated gate transistors (IGBTs), which drives the AC-DC converter fed IMD. The reference signal is maintained at 50 Hz for each modulation technique and a 10 kHz triangle signal is being utilized as the carrier signal to produce the gate pulses. Table 1 depicts the mathematical expression for three PWM methods.

**Table 1:** Mathematical representation of various PWM methods.

PWM strategy	Expression
SPWM [26]	**Table:** $I_{1}=A\sin\left(\omega t+\theta \right)$ $\left[\begin{array}{ccc} I_{a1}\; & I_{b1}\; & I_{c1} \end{array}\right]=\left[\begin{array}{ccc} I_{2_{\theta =0{}^{\circ}}}\; & I_{2_{\theta =-120{}^{\circ}}}\; & I_{2_{\theta =120{}^{\circ}}} \end{array}\right]$
THSDPWM [26]	**Table:** *X*_1_ = *I*_1_ $\begin{array}{c} =0.76A\;(\mathrm{when}\;I_{1}>0.76A)\\ =-0.76A\;(\mathrm{when}\;I_{1}<-0.76A) \end{array}$ *I*_4_ = *X*_1_ = *B* $\left[\begin{array}{ccc} I_{4a}\; & I_{4b}\; & I_{4c} \end{array}\right]=\left[\begin{array}{ccc} I_{4_{\theta =0{}^{\circ}}}\; & I_{4_{\theta =-120{}^{\circ}}}\; & I_{4_{\theta =120{}^{\circ}}} \end{array}\right]$
CSPWM [26]	**Table:** $I_{3}=\frac{2}{\sqrt{3}}\left[A\sin\left(\omega t+\theta \right)\right]-\frac{1}{2}\left\{\max\left(I_{a1},I_{b1},I_{c1}\right)+\min\left(I_{a1},I_{b1},I_{c1}\right)\right\}$ $\left[\begin{array}{ccc} I_{a3}\; & I_{b3}\; & I_{c3} \end{array}\right]=\left[\begin{array}{ccc} I_{3_{\theta =0{}^{\circ}}}\; & I_{3_{\theta =-120{}^{\circ}}}\; & I_{3_{\theta =120{}^{\circ}}} \end{array}\right]$

Table 2 shows the comparison of different power quality parameters of AC-DC converter fed IMD for various PWM techniques. For 6-pulse converter, the THD of input current is 27.11% for SPWM whereas the THD for THSDPWM and CSPWM are 27.45% and 28.02% respectively. While for the 12-pulse converter, the THD of line current is 7.37%, 6.41%, and 6.32% for SPWM, THSDPWM, and CSPWM correspondingly. Similarly, for 18-pulse converter, 4.23%, 4.99%, and 5.12% are the THD of input current for corresponding three modulation techniques.

**Table 2:** Comparison of different power quality parameters of various converters for three types of PWM techniques.

		Performance Parameters				
Pulse number of converters	PWM technique	THD of V_s_ (%)	THD of I_s_ (%)	DF	PF	V_dc_ (V)
6-pulse	SPWM	5.21	27.11	0.984	0.9654	471.9
	THSDPWM	5.69	27.45	0.987	0.9661	453.2
	CSPWM	5.82	28.02	0.991	0.9678	437.8


12-pulse	SPWM	3.11	7.37	0.9821	0.9766	532.8
	THSDPWM	3.78	6.41	0.9863	0.9679	519.3
	CSPWM	3.52	6.32	0.9905	0.9782	511.1


18-pulse	SPWM	2.97	4.23	0.9871	0.9546	561.4
	THSDPWM	2.87	4.99	0.9887	0.9678	549.4
	CSPWM	2.98	5.12	0.9956	0.9799	536.7


24-pulse	SPWM	2.59	3.21	0.9815	0.9998	577.8
	THSDPWM	2.60	3.30	0.9827	0.9997	569.1
	CSPWM	2.72	3.46	0.9968	0.9995	567.3

## 4 Performance analysis of induction motor drives

The multi-pulse rectifier removes most of the deadly odd harmonics from the input current by utilizing the phase shifting transformers which ensures the better performance of IMD.

### 4.1 Supply current profile

**Figure 6:**
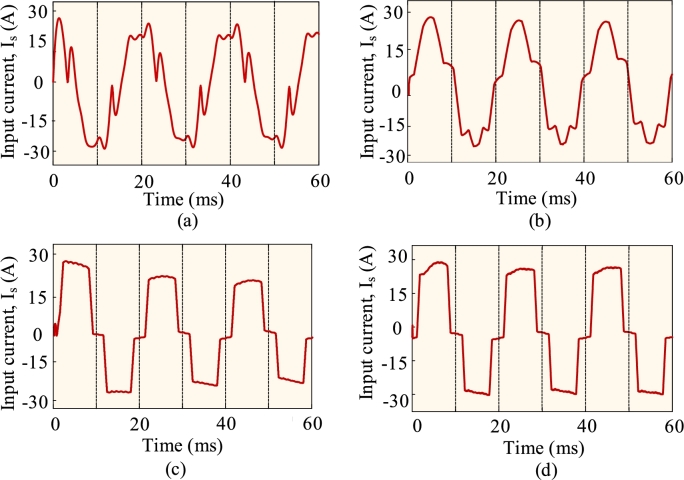
Supply current waveform of (a) 6-pulse, (b) 12-pulse, (c) 18-pulse, and (d) 24-pulse AC-DC converter fed IMD.

**Figure 7:**
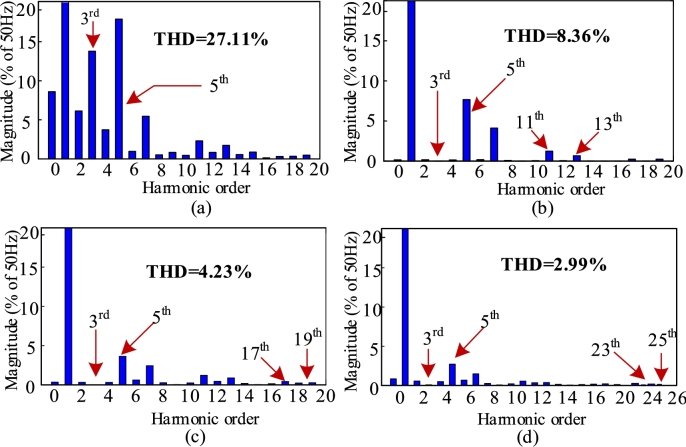
Supply current THD of (a) 6-pulse, (b) 12-pulse, (c) 18-pulse, and (d) 24-pulse AC-DC converter fed IMDs.

Fig. 6(a), 6(b), 6(c), and 6(d) depict the waveforms of supply current for 6-pulse, 12-pulse, 18-pulse, and 24-pulse AC-DC converter-fed IMDs. Accordingly, the harmonic spectrum of line current of 6-pulse, 12-pulse, 18-pulse, and 24-pulse converter-based IMDs are shown in Fig. 7(a), 7(b), 7(c), and 7(d), respectively. The THD of supply current of 6-pulse is depicted in Fig. 7(a) which is found as 27.11%. The odd harmonics including the 3^rd^ and 5^th^ order harmonics remain in the system which degrades the performance of induction motor. The THD of 12-pulse converter is found as 8.36% which is shown in 7(b). Though, the dominant odd harmonics are removed, the 11^th^ and 13^th^ order harmonics are retained. However, the supply current THD of 12-pulse converter doesn't meet the IEEE-519 standard requirement.

As a result, in order to use this converter in industry, additional procedures are required. Similarly, the 18-pulse converter eliminates the majority of fundamental harmonics, hence lowering the supply current THD to 4.23% which is shown in Fig. 7(c). However, the 17^th^ and 19^th^ order harmonics are the remaining and they are the dominating harmonics in an 18-pulse converter. Likewise, the 24-pulse converter offers 2.99% THD by taking advantage of four phase shifting transformers which is presented in Fig. 7(d).

### 4.2 Dynamic response of IMDs

**Figure 8:**
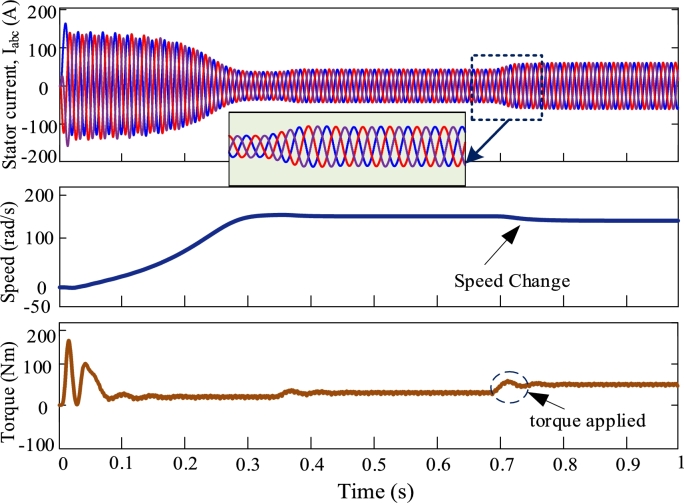
Dynamic response of 6-pulse AC-DC converter fed IMD.

**Figure 9:**
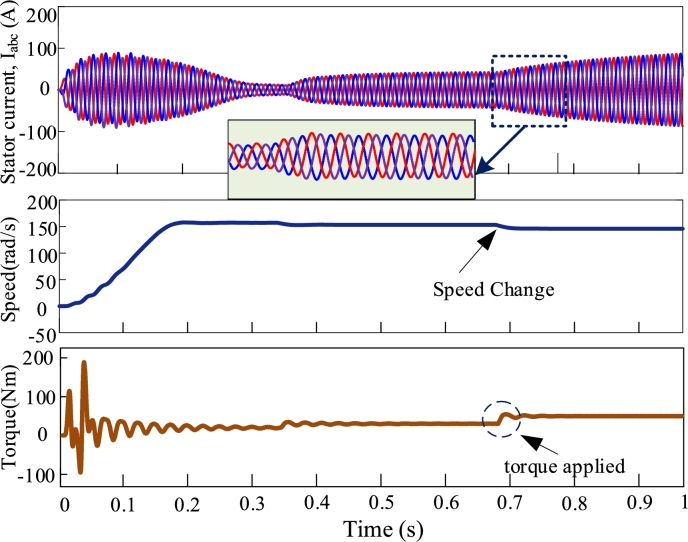
Dynamic response of 12-pulse AC-DC converter fed IMD.

**Figure 10:**
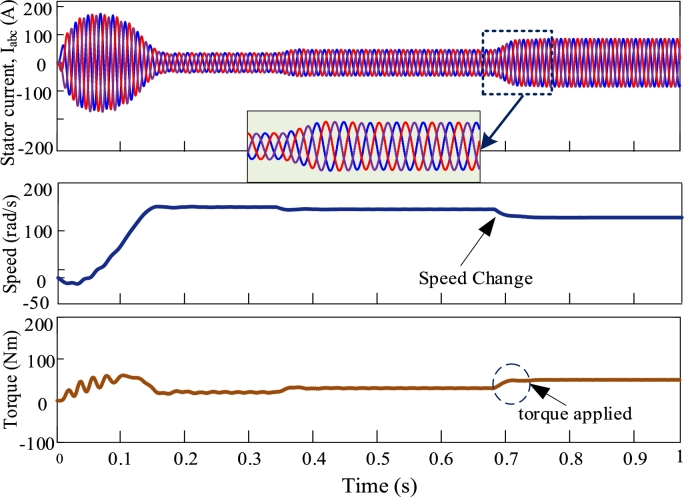
Dynamic response of 18-pulse AC-DC converter fed IMD.

**Figure 11:**
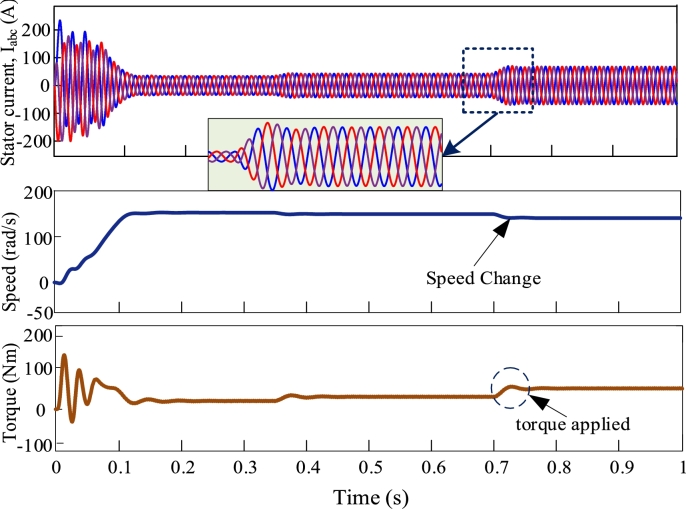
Dynamic response of 24-pulse AC-DC converter fed IMD.

Figure 8, Figure 9, Figure 10, Figure 11 illustrate the dynamic performance of induction motor at different load disturbance for four multi-pulse converters. The dynamic features of the IMDs can be classified into three categories. They are as follows: (i) stator current change, (ii) change of speed, and (iii) change of load. At t = 0.35 s and t = 0.7 s, 70% and 90% of rated torques are applied. The dynamic response of IMD for 6-pulse converter is depicted in Fig. 8. The zoomed view shows the dynamic change of stator current at the instant of applying torque. The dynamic response of 12-pulse converter fed IMD is shown in Fig. 9. For a 12-pulse converter, the rated speed drops when torque is applied and then it regains when the load is withdrawn. Likewise, 18-pulse converter fed AC-DC converter is depicted in Fig. 10. In addition, the 24-pulse converter offers smooth change in stator current and reduced THD among the other converters, as shown in Fig. 11.

## 5 Comparison and discussion

Performance parameters of IMD in terms of load variation for different multi-pulse converters are summarized in Table 3. To calculate power quality parameters such as THD of AC mains, distortion factor (DF), and power factor (PF), two types of load are considered which are full load and light load. The line current THD offered by 6-pulse and 12-pulse converters at full load are 27.11% and 7.37% respectively which doesn't meet the standard IEEE-519 criteria. On the contrary, 18-pulse and 24-pulse converters provide line current THD of 4.23% and 3.21% at full load.

**Table 3:** Comparative analysis of input power quality parameters for various converters used in IMD.

	THD of V_S_ (%)		THD of I_S_ (%)		DF		PF		
Pulse number of converters	*FL (100%)*	*LL (30%)*	*FL (100%)*	*LL (30%)*	*FL (100%)*	*LL (30%)*	*FL (100%)*	*LL (30%)*	DC-link, V_dc_ (V)
6-Pulse	5.21	3.34	27.11	55.79	0.984	1.09	0.9456	0.957	678.10
12-Pulse	3.11	1.21	7.37	9.21	0.982	0.965	0.9813	0.907	
18-Pulse	2.97	1.56	4.23	6.99	0.9871	0.955	0.9546	0.912	
24-Pulse	2.59	1.49	3.21	5.01	0.9815	0.9875	0.9998	0.933	

The THD in AC mains is lowest for 24-pulse converter. Thus it is decided that, with the increasing of pulse numbers, the power quality indicators are also increased. Lower order harmonics are extremely detrimental to the IMD as it degrades the performance. The harmonics mitigation capability of different multi-pulse converters in term of load variation is presented in Table 4. The load variation has a significant impact on the line current THD. If the load increases, it draws more current from the AC mains, hence lowering the THD.

**Table 4:** Load variation effect on supply current THD.

Load (%)	6-Pulse converter		12-Pulse converter		18-Pulse converter		24-Pulse converter	
	*I*_*s*_(*A*)	*THD of I*_*s*_*(%)*	*I*_*s*_(*A*)	*THD of I*_*s*_*(%)*	*I*_*s*_(*A*)	*THD of I*_*s*_*(%)*	*I*_*s*_(*A*)	*THD of I*_*s*_*(%)*
10	4.35	65.34	14.6	17.56	3.75	8.96	3.21	5.98
20	5.62	60.22	19.54	11.96	4.11	7.21	5.91	5.56
30	7.41	55.79	24.7	9.21	6.43	6.99	5.21	5.01
40	8.10	47.91	28.78	9.78	8.96	6.10	6.98	4.55
50	9.21	40.56	32.57	8.21	11.2	5.21	7.56	3.21

When the load increases gradually, the input current magnitude also increases which in turn reduces the THD, as depicted in Table 4. A magnitude of input current of 32.57A is recorded for the 12-pulse converter with a THD of 8.21% at 50% load. However, THD decreases slightly when the load increases, but it still falls short to IEEE-519 requirements. Similarly, for 18-pulse and 24-pulse converters, the input current is found as 11.2A and 7.56A with the corresponding THD of 5.21% and 3.21% at 50% load, as shown in Table 4. Thus, it can be concluded that the input current THD of 18-pulse and 24-pulse converter comply with the IEEE-519 standard whereas the 6-pulse and 12-pulse converter do not comply with it.

## 6 Conclusion

In this work, an analytical survey on multi-pulse AC-DC converter based IMD has been carried by highlighting the input power quality profile. Apart from input current analysis, the dynamic responses of 6-pulse, 12-pulse, 18-pulse, and 24-pulse converters are also incorporated in the work. With the increase of the pulse number of multi-pulse AC-DC converter, the THD of the input current is decreased significantly. On the contrary, the transformer size and complexity of the AC-DC power converter is increased for higher pulse converter. Different load variation effects have also been studied while calculating input current and it's THD. The input current THDs for 6-pulse and 12-pulse converters are recorded as 27.11% and 8.36%, respectively which do not comply IEEE-519 standard. Whereas the input current THDs of 4.23% and 2.99% are recorded for 18-pulse and 24-pulse system which follow IEEE-519 standard. From this survey, one can easily understand the ins and out of input power quality profile of IMD for various structures of multi-pulse AC-DC converter.

## Declarations

### Author contribution statement

Nazmul Islam Nahin: Conceived and designed the experiments; Contributed reagents, materials, analysis tools or data; Wrote the paper.

Mahdee Nafis: Conceived and designed the experiments.

Shuvra Prokash Biswas: Conceived and designed the experiments; Performed the experiments; Contributed reagents, materials, analysis tools or data; Wrote the paper.

Md. Kamal Hosain: Performed the experiments; Wrote the paper.

Pranta Das; Safa Haq: Analyzed and interpreted the data; Contributed reagents, materials, analysis tools or data.

### Funding statement

This research did not receive any specific grant from funding agencies in the public, commercial, or not-for-profit sectors.

### Data availability statement

Data will be made available on request.

### Declaration of interests statement

The authors declare no conflict of interest.

### Additional information

No additional information is available for this paper.

## Appendix A

The Motor specifications: Three-phase squirrel-cage induction motor, 7.2 hp (5.4 kW), three phase four pole, Y connected, 415 V, 50 Hz.

Line filter: $R_{s}=2.5\;\Omega$, $L_{s}=2$ mH.

DC link filter: $L_{d}=2$ mH, $C_{d}=3200\;\mu \text{F}$.

Inverter filter: $L_{m}=2$ mH, $C_{m}=3.3\;\mu \text{F}$.
